# The Evaluation of Renal Iron Deposition With a 3 Tesla MRI Device in Beta-Thalassemia Major Patients

**DOI:** 10.7759/cureus.36179

**Published:** 2023-03-15

**Authors:** Tevfik Guzelbey, Zeynep Ece Demirbaş, Bengi Gurses

**Affiliations:** 1 Department of Interventional Radiology, Istanbul Başakşehir Çam and Sakura City Hospital, Istanbul, TUR; 2 Department of Internal Medicine, Dr. Siyami Ersek Cardiovascular and Thoracic Surgery Education and Research Hospital, Istanbul, TUR; 3 Department of Radiology, Koc University School of Medicine, Istanbul, TUR

**Keywords:** 3-tesla mri, serum ferritin, iron overload, mri, beta thalassemia major

## Abstract

Background and objective

Beta-thalassemia is the most frequent monogenic disease in the world. In beta-thalassemia major (BTM) patients, blood transfusions for severe anemia usually cause iron overload, leading to increased morbidity and mortality. In this study, we aimed to examine the iron overload in the kidneys of BTM patients with a 3 Tesla (3T) MRI device and assess the relationship between iron overload in the liver and heart as well as serum ferritin levels.

Methods

This was a retrospective study covering the period between November 2014 and March 2015. MRI was performed on 21 patients with BTM who were receiving blood transfusions and chelation therapy. The control group (n=11) included healthy volunteers. A 3T MRI device (Ingenia, Philips, Best, The Netherlands) using a 16-channel phased array SENSE-compatible torso coil was used. Three-point DIXON (mDIXON) sequence and the relaxometry method were employed to measure iron overload. Both kidneys were analyzed via mDIXON sequence for atrophy or variations. Afterward, the images in which renal parenchyma could be distinguished best were selected. Iron deposition was analyzed via the relaxometry method using a unique software (CMR Tools, London, UK). All data were analyzed using IBM SPSS Statistics v.21 (IBM Corp., Armonk, NY). The Kolmogorov-Smirnov test, independent samples t-test, Mann-Whitney U test, and Pearson’s and Spearman’s rho correlation coefficient were used. A p-value <0.05 was considered statistically significant.

Results

There was a statistically significant relationship between beta-thalassemia patients who had cardiac iron deposition and those who did not in terms of T2* time (p=0.02). In contrast, there was no similar relationship for liver iron deposition (p>0.05). Renal T2* values were significantly different between the patient and control groups (p=0.029). T2* times were significantly different between patients who had ferritin levels below 2500 ng/ml and those with ferritin levels above 2500 ng/ml (p=0.042).

Conclusion

Based on our findings, 3T MRI is a safe and reliable tool for screening iron overload in BTM patients as it makes distinguishing between renal parenchyma and renal sinus much easier and as it is more sensitive to iron deposition.

## Introduction

Iron is the most important component of vital proteins like hemoglobin and myoglobin. However, iron overload causes organ dysfunction via the accumulation of oxygen and hydroxyl radicals in the organelles of the cells [1]. In severe chronic anemia patients like those with beta-thalassemia, frequent blood transfusion leads to iron deposition despite chelation therapy. Besides, the most important factor associated with morbidity and mortality in these patients is organ dysfunction due to iron deposition [2].

Beta-thalassemia, the most frequent monogenic disease in the world, is inherited autosomal recessively. Because both the beta globulin genes are affected, beta globulin chain production is damaged and this causes chronic severe anemia in beta-thalassemia major (BTM) patients [3]. Hemosiderin accumulation is demonstrated in postmortem glomerular cells [4]. In patients who have iron deposition, proximal tubular dysfunction is seen and this is thought to be due to iron accumulation and also oxidative stress of chronic anemia [5]. Furthermore, chelation therapies could additionally cause proximal tubular dysfunction [6].

In terms of life expectancy and quality, early diagnosis and prevention of renal failure are critical. Renal function tests are crucial for early diagnosis but they do not reveal information about pathophysiology. Renal biopsy, on the other hand, is an invasive procedure and has a markedly high bleeding risk, and cannot be repeated consecutively [7]. Multi-echo gradient (MEGRE) T2* weighted MRI is a reusable, non-invasive, and safe technic that is used in liver and renal iron deposition imaging and its accuracy has been proven by biopsies [8,9]. Despite the scarcity of studies on the topic, MEGRE evaluation of other organs like the pancreas, spleen, pituitary and adrenal glands, and bone marrow has been described [10-12].

All studies about renal iron depositions in the literature so far have used 1.5 Tesla (1.5T) MRIs [13-16] and to the best of our knowledge, there are no published studies involving 3 Tesla (3T) MRIs. In light of this, we aimed to measure renal iron overload in beta-thalassemia patients and healthy controls with MEGRE T2* sequence in a 3T MRI device and asses the relationship between iron overload in the liver and heart and compare the data with ferritin levels.

## Materials and methods

Patient population

All data were collected from the hospital records of beta-thalassemia patients presenting to our hospital between November 2014 and March 2015. Patient consent forms and ethics committee approval were obtained. A total of 21 BTM patients periodically getting blood transfusions were enrolled in our study. One patient was excluded owing to concerns about the possibility of unreliable measurement due to an atrophic right kidney. All patients had normal creatinine levels and none had a history of chronic illness except for BTM. The mean age of 20 patients (eight females and 12 males) enrolled in our study was 28.6 ± 6.92 years. All patients were getting blood transfusions every two to four weeks apart from regular chelation therapy. According to patient compliance and their ferritin levels, they were taking mono or combination therapy of oral deferasirox (20-40 mg/kg/d), oral deferiprone (75-100 mg/kg/d), and subcutaneous deferoxamine (40 mg/kg/d). MRI was performed one week before the blood transfusion. Plasma ferritin levels were measured up to two weeks after the blood transfusion and one week before the MRI. The control group included 11 volunteers (six males and five females). The mean age of the controls was 32.4 ± 5.92 years (range: 22-41 years). None of the volunteers had a history of chronic illness, blood transfusion, or chronic kidney disease. The exclusion criteria for our study were as follows: symptomatic renal, cardiac, or liver diseases; hypertension; diabetes mellitus; other chronic illnesses and claustrophobia.

Magnetic resonance imaging

All imaging procedures were done with a 3T MRI device (Ingenia, Philips, Best, The Netherlands) using a 16-channel phased array SENSE-compatible torso coil. During the procedure, supine-positioned patients were asked to hold their breaths. Throughout the cardiac imaging procedure, ECG and respiratory pads were used if required for triggering.

mDIXON sequence was used to determine the localization and size of the kidneys, or structural anomalies if they existed; 16 echo times (in the range of 2.1-2.5 ms), fat-suppressed, breath-hold, gradient turbo field echo sequence was used to evaluate the iron deposition in the kidneys and liver. To determine the cardiac iron deposition, the ECG-triggered, breath-hold, black-blood, fat-suppressed, midventricular short axis MEGRE T2* sequence was used as per Wood et al. [17,18]. The information on the sequence is presented in Table 1.

**Table 1:** Sequence information gathered to analyze kidney, liver, and cardiac iron deposition FOV: field of view; MEGRE: multi-echo gradient; NSA: number of signals averaged; TE: echo time; TR: repetition time

	Kidney and liver MEGRE sequence	Cardiac MEGRE sequence
TR	28 ms	28 ms
TE	Between 2.1-25 ms and 16 echo	Between 2.1-25 ms and 16 echo
Flip angle	20°	20°
FOV	370 mm	350 mm
Matrix	168 x 320	146 x 288
Slice thickness	5 mm	10 mm
NSA	2	1
Turbo factor	144	128

Analyzing the images

Firstly, both kidneys were analyzed via the mDIXON sequence for atrophy or variations. Afterward, the images in which renal parenchyma could be distinguished best were selected. Iron deposition was analyzed via the relaxometry method using a unique software (CMR Tools, London, UK).

**Figure 1:**
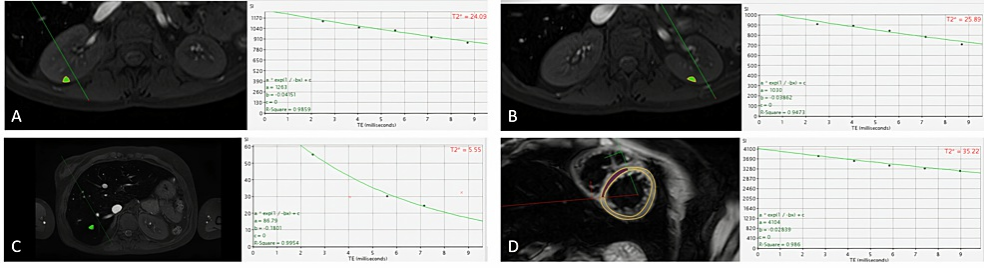
T2* time curves and ROIs drawn for T2* time of the right kidney (A), left kidney (B), liver (C), and heart (D) respectively in a sample patient ROI: region of interest

Based on Grassedonia et al.'s [13] finding that there was no difference in the measurements between distinct areas of the kidney, our measurements were performed using region of interest (ROI) drawing for the sites of best renal parenchymal images, by paying attention to motion artifacts. For evaluating liver iron deposition, measurements were performed by calculating the average after drawing three ROIs, one on the right and two on the left lobes of the liver, far from the motion artifact areas. Liver T2* values were converted into liver iron concentration (LIC) values using the formula proposed by Wood et al. [8]. The cardiac iron deposition was measured by ROI drawn on the area including the interventricular septum, endo, and epicardium (Figure 1).

When adapting the cut-off values from 1.5T to 3T, the formula proposed by Storey et al. was used [19]. The cut-off value for LIC was determined as 3.3 ms. The values were classified as follows - mild: between 1.4 and 3.3 ms, moderate: between 0.7 and 1.4 ms, and severe: below 0.7 ms. The cut-off value for cardiac iron concentration was determined as 12 ms. The values were classified as follows - mild: between 7.8 and 12 ms, moderate: between 5.7 and 7.8 ms, and severe: below 5.4 ms.

Statistical analyses

All data were analyzed using IBM SPSS Statistics v.21 (IBM Corp., Armonk, NY). Continuous variables were presented using mean and standard derivation (SD) or median and interquartile range (IQR; 75th and 25th percentiles). The convenience of the data to normal distribution was evaluated by the Kolmogorov-Smirnov test. In the presence of two independent groups for comparison, independent samples t-test and Mann-Whitney U test were used. In order to analyze whether there was a correlation between the two variables, Pearson’s correlation coefficient in a normal distribution and Spearman’s rho correlation coefficient in abnormal distribution were employed.

## Results

Laboratory findings and MRI parameters of BTM patients and the control group are presented in Table 2.

**Table 2:** MRI parameters and laboratory data of control and patient groups IQR: interquartile range; LIC: liver iron concentration; MRI: magnetic resonance imaging; SD: standard deviation

	Patients (n=20)	Controls (n=11)	P-value
Males/females, n (%)	8/12 (40/60%)	6/5 (54/46%)	
Age, years, median (IQR)	27 (22.25-33)	32.00 (29-38)	0.104
Right renal T2*, ms, mean ± SD	41.17 ± 17.52	57.90 ± 5.58	0.03
Left renal T2*, ms, mean ± SD	43.39 ± 20.44	57.20 ± 7,52	0.09
Cardiac T2*, ms, median (IQR)	23.13 (8.34-27.07)	26,39 (24.38-27.64)	0.095
Liver T2*, ms, median (IQR)	2.14 (1.39-5.31)	20.16 (17.61-22.27)	˂0.001
LIC, mg/g liver dry weight, median (IQR)	6.53 (2.74-9.51)	0.97 (0.91-1.06)	˂0.001
Serum ferritin, µg/L, median (IQR)	1016 (567-2349)	39 (21-67)	˂0.001

**Figure 2:**
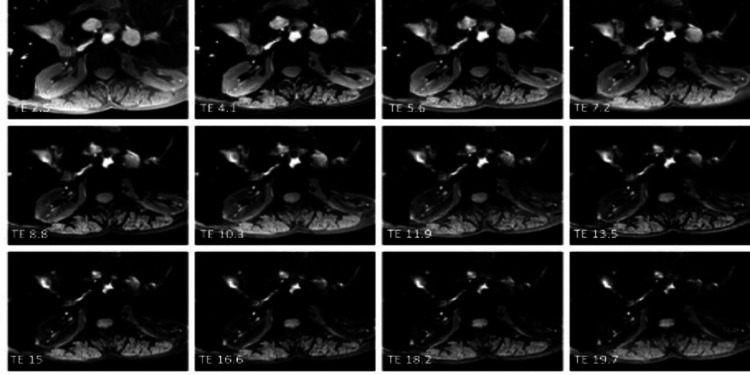
An example of a BTM patient’s multi-echo gradient T2* sequence images As TE increases due to iron overload, marked signal loss can be seen in the liver and kidneys BTM: beta-thalassemia major; TE: echo time

The average T2* time was 41.17 ± 17.52 for the right kidney and 43.39 ± 20.44 ms for the left kidney in the patient group (Figure 2).

**Figure 3:**
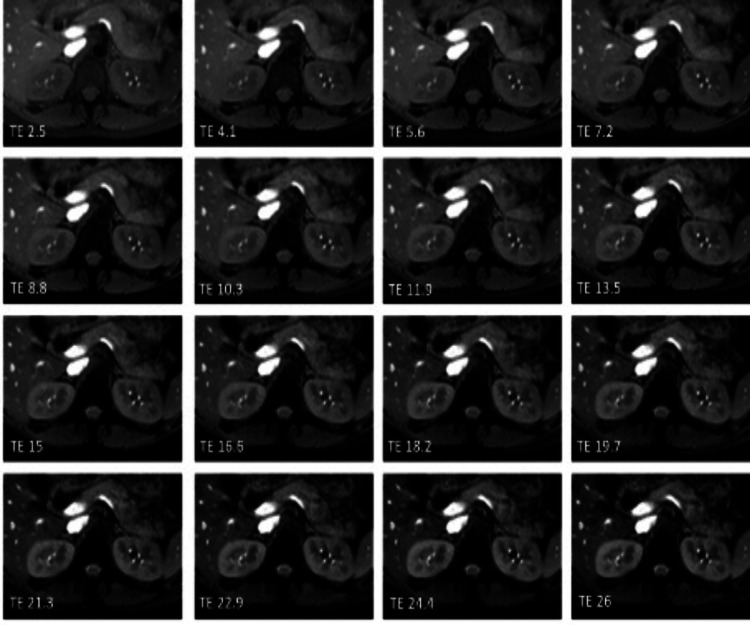
An example of the control group multi-echo gradient T2* sequence images As there is no excess iron overload, at distinct TEs, signal differentiation cannot be observed in the liver and kidneys TE: echo time

In the control group, the average T2* time was 57.90 ± 5.58 for the right kidney and 57.20 ± 7.52 ms for the left kidney (Figure 3).

Renal T2* values between the patient and control groups were significantly different (p=0.029). However, there was no significant difference between the right and left kidneys in the control and patient groups (p>0.05). Therefore, comparisons with other parameters were made by calculating the average time for the right and left kidneys.

Correlation between renal T2* time and liver T2* time was weak (r=0.596, p=0.006); for the LIC value, it was negative and moderate (r=-0.600, p=0.005); for ferritin, it was moderate (r=0.517, p=0.020), and it was weak for cardiac T2* time (r=0.471, p=0.036). There was no significant correlation either between renal T2* time and liver-cardiac times or LIC levels (p>0.05). There was also no significant relationship between renal T2* time and age in both groups (p>0.05).

There was a statistically significant relationship between beta-thalassemia patients who had cardiac iron deposition and those who did not in terms of T2* time (p=0.02). In contrast, there was no relationship between the patients who had liver iron deposition and those who did not (p>0.05). T2* times were significantly different between patients who had ferritin levels below 2500 ng/ml and those with ferritin levels above 2500 ng/ml (p=0.042).

## Discussion

Increased iron overload in cells causes the Fenton reaction, which leads to free oxygen radical release. These radicals may cause damage to the structure of DNA and proteins and also lipid peroxidation [1]. This cascade induces organelle dysfunction and ultimately injury to the organ in which iron is accumulated. Iron in renal glomerular cells, oxidative stress due to chronic anemia, and iron chelators may contribute to an increase in creatinine levels and renal failure in the upcoming period [5,20]. But it is a matter of debate as to whether iron chelators increase creatinine levels via nephrotoxicity of itself or due to the damage resulting from high iron excretion [20,21].

Based on our finding that there is a significant difference in renal T2* times between beta-thalassemia patients and the control group, we can assume that iron is accumulated in kidneys and this can be demonstrated via MEGRE T2* sequence. Also, this finding can be supported by the difference in renal T2* times between the patients who had ferritin levels below 2500 ng/ml and those above 2500 ng/ml and also between patients who had cardiac iron deposition and those who did not.

According to the results of other studies in the literature, there was also no significant difference in right and left kidney T2* times between healthy volunteers and patients who had iron overload due to chronic anemia and transfusions, which aligns with our findings [13,14,16]. Due to similar reasons, they used the average T2* time of both kidneys or just used one kidney to compare with other parameters.

ElAlfy et al. [16] showed a weak correlation between renal R2* time and LIC value and a moderate negative correlation with cardiac T2* time. Hashemieh et al. [22] showed a weak correlation between renal T2* time and liver and also cardiac T2* time. While Hashemieh et al. revealed a weak correlation between ferritin and renal T2* time, ElAlfy et al. [16] could not find any correlation at all. We found a moderate correlation between renal T2* time and ferritin levels and also a weak correlation between liver T2* time and cardiac T2* time. On the other hand, Schein et al. [14] showed a significant correlation between renal, cardiac, and liver R2* values; however, they could not find any correlation in renal R2* time between the control group and thalassemia patients. They considered that renal iron toxicity might have been below the limit that could be detected via MRI and this limitation might have led to these findings. However, in our study and that of ElAlfy et al. [16], a statistically significant difference was shown in renal T2* time between the healthy control group and beta-thalassemia patients. There was no significant association between age and renal T2* time in our study, which is consistent with the literature [16,22].

In tissues like kidneys that are less prone to iron deposition, measurements done with a 3T MRI device can provide more accurate information as it is more sensitive to iron inhomogeneity. There is a linear correlation between T2* relaxation and magnetic field force. As there is less magnetic field force in 0.5T devices, T2* will be less affected and less sensitive in detecting mild iron deposition [23]. In a 1.5T magnetic field, the signal intensity of severe iron deposition is going to reach the noise level, and hence it would be hard to measure T2* relaxation time [24]. Thanks to the increased magnetic field force of 3T devices, these devices are supposed to be more sensitive in detecting mild iron deposition and less sensitive in detecting severe iron deposition than 1.5T devices. As renal iron deposition is milder than that in the heart and liver, it would be more accurate to use 3T devices for kidney imaging in thalassemia. Also, the distinction between the renal sinus and parenchyma can be defined more clearly with 3T MRI devices [25]. This feature also provides more accurate measurements.

This study has a few limitations, primarily related to its retrospective design. While early biomarkers of renal dysfunction like creatinine, cystatin c, and urinary beta-2 microglobulin are analyzed in other studies about renal T2* measurements [15,16], specific blood tests could not be performed in our study as it was designed retrospectively. Another limitation due to our study's retrospective design was not using a specific sequence adjusted for kidneys. However, kidney parenchyma can clearly be distinguished in the images taken of the liver. Our other limitations include not testing early biomarkers of kidney dysfunction, the relatively smaller sample size, and employing only one radiologist for measurements.

Finally, the effect of kidney iron deposition solely on renal dysfunction is a matter of debate. Studies involving larger and more homogeneous patient populations are required to further examine the effects of renal iron deposition on renal dysfunction as well as the effects of chelation therapy on renal iron deposition.

## Conclusions

Renal iron accumulation is known to occur as a result of excess iron load in the whole body. A 3T MRI device can provide more accurate and reliable information as it makes distinguishing between renal parenchyma and renal sinus much easier and as it is more sensitive to iron deposition. Including kidneys alongside the heart and liver and adjusting sequences during routine imaging will lead to more efficient and cost-effective procedures and help in the early recognition of renal dysfunction.
